# Axial Behavior of Reinforced UHPC-NSC Composite Column under Compression

**DOI:** 10.3390/ma13132905

**Published:** 2020-06-28

**Authors:** Fuhai Li, Yunfeng Hexiao, Hao Gao, Kailai Deng, Yilin Jiang

**Affiliations:** 1 Department of Building Material, Southwest Jiaotong University, Chengdu 610031, China; lifuhai2007@home.swjtu.edu.cn (F.L.); hxyf19681130@my.swjtu.edu.cn (Y.H.); GAOhao@my.swjtu.edu.cn (H.G.); jiangyilin2020@my.swjtu.edu.cn (Y.J.); 2 Key Laboratory of High-Speed Railway Engineering, Ministry of Education, Southwest Jiaotong University, Chengdu 610031, China; 3 Department of Bridge Engineering, Southwest Jiaotong University, Chengdu 610031, China

**Keywords:** composite column, exterior UHPC cover, compression test, strain–stress relationship, confined model

## Abstract

This paper proposes a novel reinforced ultra-high-performance concrete (UHPC)-normal strength concrete (NSC) composite column. The main feature of the reinforced UHPC-NSC composite column is the use of an exterior UHPC cover, which is helpful for improving the load-carrying capacity, deformability, and crack resistance. This study focused on the axial behavior of the reinforced UHPC-NSC composite column. A total of 12 specimens were designed to investigate the axial behavior of the composite column under compression. The thickness of the exterior UHPC cover, volumetric stirrup ratio, and construction method were considered as the main experimental parameters. The failure modes and strain–stress relationships are discussed in this paper. By introducing the exterior UHPC cover, the peak confined strength obviously improved. The compressive strain at the peak confined strength was mainly determined by the deformability of the plain UHPC material. The composite column resulted in a unique sudden drop immediately after the peak confined strength, owing to the rapid loss of the confining stress from the exterior UHPC cover. A trilinear model is proposed to describe the strain–stress relationship of the composite column. The results obtained by the modified model are in good agreement with the test results.

## 1. Introduction

Ultra-high-performance concrete (UHPC) has been extensively investigated and is widely used in practical engineering, owing to its excellent mechanical properties, such as its high elastic modulus, high compressive and tensile strength, and strain hardening in tension. Many novel components and structures consisting of UHPC have been developed, such as the steel-UHPC composite deck system, delay-casted UHPC wet joint in bridge decks, and so on [1,2,3,4]. With the application of UHPC, the crack resistance and load-carrying capacity of these components are significantly improved. However, most UHPC applications mainly focus on the material’s tensile strength and strain hardening in tension. Therefore, the advantage of high compressive strength has not been fully exploited. To extend the application of the compressive strength of UHPC, Zeng et al. (2020) investigated the compressive behavior of full-scale reinforced UHPC columns, and proposed a strain–stress model for the reinforced UHPC columns [5]. The test results revealed that the tensile strength of the UHPC material contributed the most to its confining effect, while the confining effect from the stirrup was relatively small. However, the column exhibited brittle failure even for the specimen with the volumetric ratio of 0.13, which is evidently higher than the minimum value (0.08) required by Eurocode 8 [6]. The full-section employment of UHPC significantly contributed toward complete brittle failure under compression and increased the cost of the structure. 

To balance the economic cost and mechanical performance, the partial employment of high-performance cement-based materials has attracted much attention. Hajek et al. (2012) have proposed timber-UHPC composite floor structures to improve the acoustic performance and fire safety of structures [7]. Their test results revealed that the connection between the UHPC and the timber beam is the most important factor for ensuring the performance of composite floor structures. Maalej and Leong developed an Engineered Cementitious Composite (ECC)-normal strength concrete (NSC) composite beam [8]. The use of ECC in the tensile area delayed the debonding failure between the ECC materials and the attached fiber-reinforced polymer sheet. The deformability of this composite beam was obviously larger than that of a common reinforced NSC beam. Another ECC-masonry composite wall has also attracted the attention of scholars. By introducing the ECC material into the unreinforced masonry wall, the out-of-plane resistance of masonry bricks was improved. Quasi-static testing has also been used to achieve the significant improvement of the load-carrying capacity and ductility [9]. Li et al. (2019) partially employed ECC in a column to balance the economic cost and mechanical performance [10]. By using ECC as the exterior cover, the ductility of the column significantly improved. However, the elastic modulus of ECC was too small to provide adequate confining stiffness. The cover layer requires some other material with high tensile strength and stiffness simultaneously. Caluk et al. proposed durable bridge columns using stay-in-place UHPC shells [11]. The introduction of a UHPC shell as the formwork improved the durability of the bridge column and prevented plastic hinge development at the bottom of the column. However, cyclic tests on this novel column have not been carried out yet. The abovementioned studies have demonstrated that the partial employment of high-performance cement-based materials with NSC is an effective approach toward simultaneously improving the structural performance and reducing the cost. 

This paper proposes a reinforced UHPC-NSC composite column to enhance the load-carrying capacity and resistance of earthquake-induced crack, while maintaining low cost. The composite column comprises the exterior UHPC cover, inner NSC core, and common reinforcement cage. The exterior UHPC cover is expected to simultaneously provide the axial load-carrying capacity, crack resistance, and circumferential confinement for the inner NSC core. To investigate the axial behavior of this novel column under compression, twelve specimens were designed and tested. The thickness of the exterior UHPC cover, volumetric stirrup ratio, and construction method were considered the experimental parameters. The observed failure patterns are discussed in this paper. Compared with classical confined concrete models, the composite column exhibited completely different strain–stress relationships. According to the test results, a modified confined model is proposed specifically for the reinforced UHPC-NSC composite column.

## 2. Experiment

### 2.1. Specimen Design

**Figure 1:**
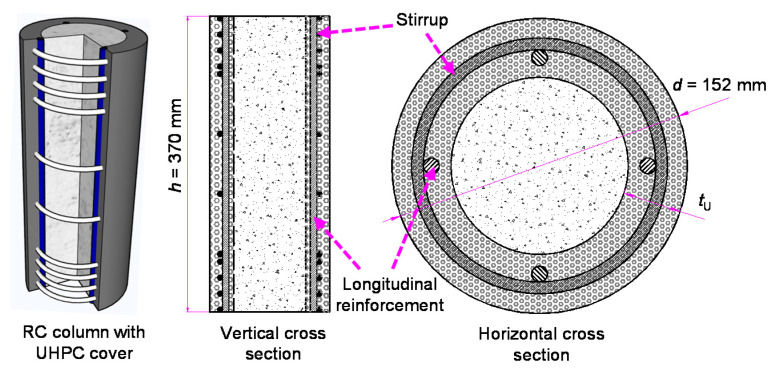
Structure of reinforced ultra-high-performance concrete (UHPC)- normal strength concrete (NSC) composite column.

The specimen of the proposed reinforced UHPC-NSC composite column is shown in Figure 1. The NSC was poured into the inner core, and the UHPC was used as the exterior cover. The longitudinal reinforcement and stirrup were the same as those of a common reinforced column. With an exterior UHPC cover, the column is expected to have a larger load-carrying capacity and better crack resistance. The spacing of stirrup at the two ends of the specimen was 20 mm, to ensure that failure occurred at the middle area of the column. For different specimens, the height of the strengthened area was different to match the designated stirrup spacing in the middle area.

**Figure 2:**
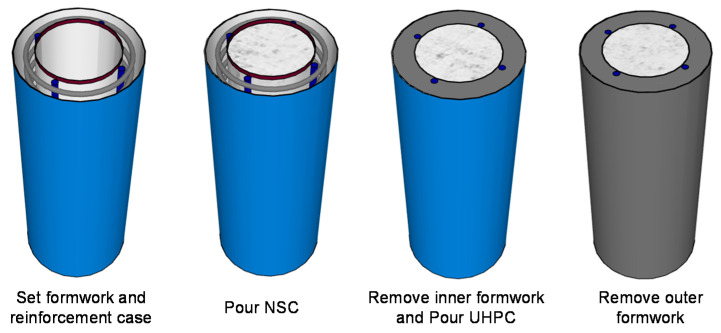
Construction process of reinforced UHPC-NSC composite column.

The construction process of the composite column is shown in Figure 2. First, the two layers of formwork and the reinforcement case were set. Then, the inner NSC core was poured. After the initial setting of NSC, which is typically two days, the inner formwork was removed, and the exterior UHPC was poured. Finally, the outer formwork was removed. Obviously, the pouring sequence for the inner NSC core and exterior UHPC cover could be reserved, and this reversibility was considered one of the experimental parameters.

In total, 12 specimens were designed as presented in Table 1, including one reinforced NSC column, one reinforced UHPC column, and ten reinforced composite columns. The diameter, height and longitudinal reinforcement were the same for all specimens: 152 mm, 370 mm, and four HRB400 rebar with the diameter of 8 mm, respectively.

In this study, the thickness of the exterior UHPC cover, volumetric stirrup ratio, and construction method were considered as the main parameters. Moreover, ‘U*x*-GJ*y*’ indicates that the specimens had a UHPC cover with x-mm thickness and y-mm spacing between the adjacent stirrups. Note that the spacing of stirrup varies from 75 mm to 200 mm, which is too large to provide adequate confining effect. For the small scaled columns, the diameter of stirrup could not be reduced. Thus, the spacing of the stirrup will increase, to maintain the similar volumetric stirrup ratio with practical columns. In further tests, attention should be paid to the influence of size effect on specimen design. Additionally, ‘U*x*-GJ0′ indicates that no stirrup was used in the specimens, even in the redoubled area. Finally, ‘U*x*-GJ*y*-L’ indicates that the inner NSC core was poured after the initial setting of the exterior UHPC cover. There was only one sample for each type of specimen. As a proof-of-concept study, the main objective was to choose the most relevant characteristics and make a detailed research plan with fewer parameters and higher number of samples.

### 2.2. Material Properties

**Figure 3:**
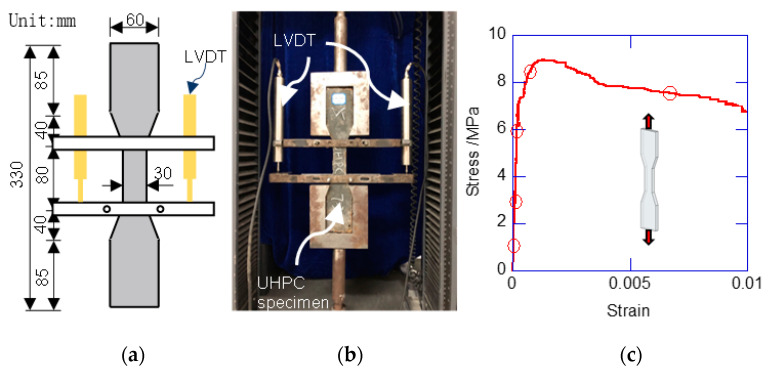
Tensile test of UHPC. (**a**) Dimensions of UHPC dumbbell specimens; (**b**) loading and monitoring setup; (**c**) tensile stress–strain curve of UHPC.

The UHPC ingredients used in the test are listed in Table 2. Figure 3 shows the tensile test for the UHPC. The dumbbell specimens were fabricated and tested. The UHPC material exhibited a quasi-ductile failure mode. Before the initial cracking, the stress linearly developed to the initial cracking strength. After the initial cracking, the UHPC exhibited slight strain hardening. The ultimate tensile strength appeared at the strain of 1.08 × 10^−3^, at approximately 9.29 MPa. Then, the stress started to decrease with a relatively slow rate. After the tensile strain exceeded 0.01, the tensile stress rapidly decreased. Compressive tests were conducted using the prism specimen with a size of 100 mm × 100 mm × 300 mm. Under compression, the UHPC exhibited brittle failure. Table 3 lists the mechanical properties of the UHPC and NSC. Compared with the NSC, the UHPC had a much higher elastic modulus, compressive strength, and strain at peak strength. The yielding stresses of the 6-mm stirrup and 8-mm longitudinal rebar were 520 MPa and 451 MPa, respectively.

### 2.3. Loading and Measurement System

**Figure 4:**
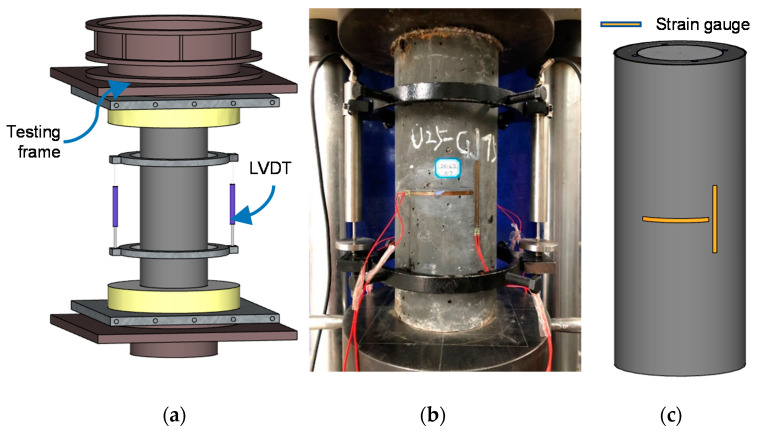
Loading setup and measurement system. (**a**) 3D view of the loading setup; (**b**) test setup of reinforced UHPC-NSC composite column; (**c**) layout of strain gauges.

The loading setup and measurement system is shown in Figure 4. A displacement-controlled compression testing machine was used. In the compression test, the strain rate was set to 2.3 × 10^−5^ /s according to the requirements specified by the Chinese concrete strength standards [12]. The axial load was applied on both UHPC cover and NSC core. Two linear variable differential transformers (LVDTs) were used to monitor the specimens’ axial deformation. Four strain gauges were pasted at the middle of the height of each specimen to measure the vertical compression and circumferential expansion.

## 3. Results

### 3.1. Damage and Failure Mode

**Figure 5:**
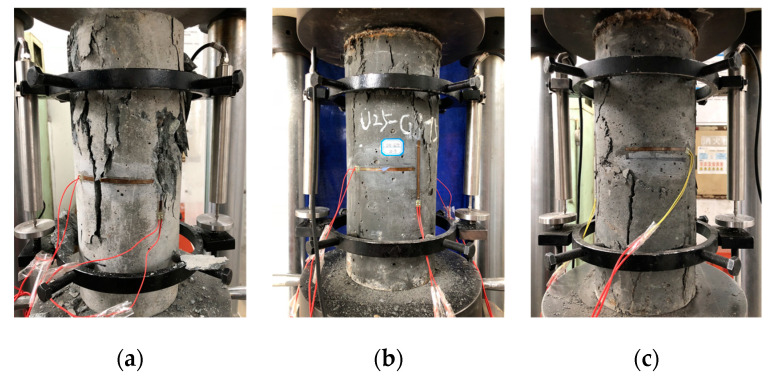
Failure mode of representative specimens. (**a**) U0-GJ75; (**b**) U25-GJ75; (**c**) U76-GJ75.

The failure modes of U0-GJ75, U25-GJ75, and U76-GJ75 are shown in Figure 5. The reinforced NSC column exhibited similar failure modes as those observed in many previous studies. The vertical crack appeared in the middle area, and gradually developed through the entirety of the specimens. Additionally, the specimens lost their load-carrying capacity owing to the large-scale spalling of NSC. The reinforced UHPC column exhibited brittle failure soon after achieving its peak load, which is consistent with the test results by Zeng et al. (2020) [5]. For the composite column, the initial crack occurred on the UHPC surface at the peak axial load. Immediately after the peak load, the crack vertically run through the exterior UHPC cover. Unlike the reinforced UHPC column, the composite column did not completely lose its load-carrying capacity after the vertical crack penetration. Compared with the reinforced UHPC column, the crack of the thin UHPC cover could not instantly release excessive energy, and the stirrup maintained the confinement for the core area. Thus, owing to the confinement from the stirrup, the composite column could still carry a certain axial load. However, the load-carrying capacity obviously decreased.

**Figure 6:**
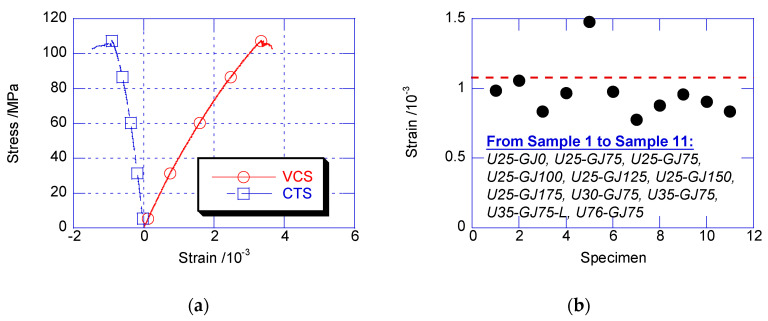
Strain development on UHPC surface. (**a**) Strain development; (**b**) circumferential tensile strain (CTS) at peak confined strength.

The circumferential tensile strain (CTS) and vertical compressive strain (VCS) of U35-GJ75 are compared in Figure 6a, wherein the compressive strain is shown to be positive. Before achieving the peak confined stress, the CTS developed proportionally with the VCS. This result reveals that there was good linearity before the peak confined stress. At the peak confined stress, the CTS of U35-GJ75 was 0.98 × 10^−3^, which is close to the ultimate tensile strain of the plain UHPC. Figure 6b shows the CTS at the peak confined stresses for all the composite columns, which was close to the ultimate tensile strain. For a cylinder consisting of a material with tension strength, which was much less than the compression strength, the essence of compressive failure is that the circumferential tensile stress at the edge achieves tension strength [5,13]. This conclusion is also supported by the CTS development in the composite column at peak strength.

### 3.2. Strain–Stress Relationship

In this experiment, it was difficult to distinguish the load sharing of the exterior UHPC cover and inner NSC core. Moreover, the exterior UHPC cover also underwent two different mechanical conditions, namely, a condition of being confined by the stirrup and a condition of not being confined by the stirrup. Thus, this study employed the average confined stress of the area surrounded by the central axis of the stirrup, as discussed below.

The average confined stress was calculated using Equation (1), where *F* is the total axial load; *F*_cov_ is the load shared by the 14-mm thick cover layer consisting of UHPC; *F*_s_ is the load carried by the longitudinal reinforcement, *A*_core_ is the sectional area surrounded by the axis line of the stirrup. *F*_cov_ can be obtained by interpolation, as expressed in Equation (2); $\sigma_{u}$ is the compressive stress of the plain UHPC. Here, *F*_s_ conforms to the bilinear development expressed in Equation (3).

(1) $$\sigma_{\mathrm{cc}}(\varepsilon )=\frac{F(\varepsilon )-F_{\mathrm{cov}}(\varepsilon )-F_{s}(\varepsilon )}{A_{\mathrm{co}\mathrm{re}}}$$

(2) $$F_{\mathrm{cov}}(\varepsilon )=\sigma_{u}(\varepsilon )A_{\mathrm{cov}}$$

(3) $$F_{s}(\varepsilon )\left\{\begin{array}{ll} A_{s}E_{s}\varepsilon & \varepsilon \leq \varepsilon_{y}\\ A_{s}f_{y} & \varepsilon >\varepsilon_{y} \end{array}\right\}$$

**Figure 7:**
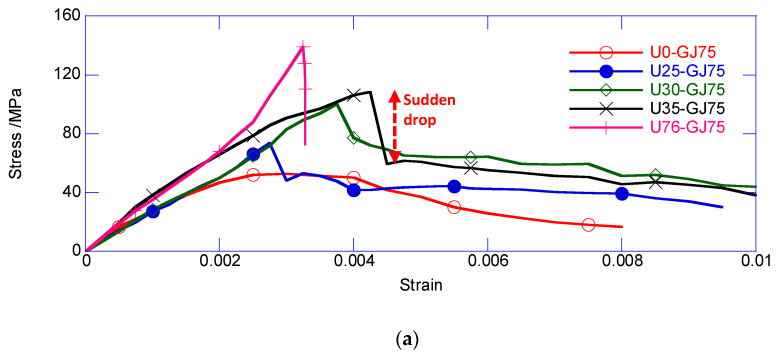

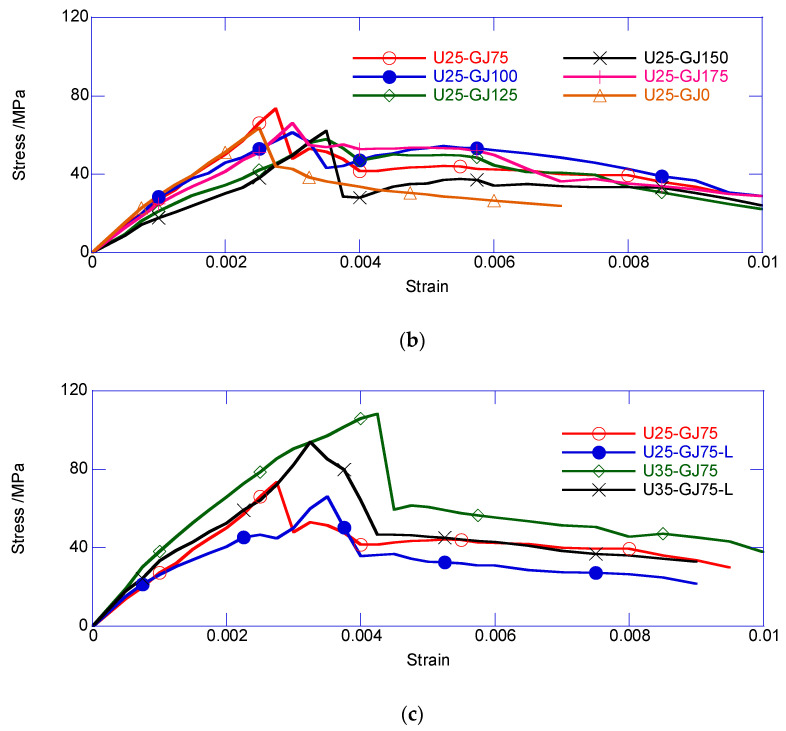
Strain–stress relationship of confined core area. (**a**) Effect of thickness of exterior UHPC cover; (**b**) effect of volumetric stirrup ratio; (**c**) effect of construction method.

The strain–stress relationship of the area confined by the stirrup is shown in Figure 7. Generally, the confined stress of the composite column underwent a sudden decrease, which is a unique feature, and also the most important feature. Immediately after the peak confined strength, a vertical crack occurred on the exterior of the UHPC cover, and eliminated the confinement from the exterior UHPC cover. Thus, the sudden loss in the confining stress resulted in the sudden decrease of the confined stress.

Figure 7a compares the effects observed with different thicknesses for the exterior UHPC cover. The initial stiffness of the five specimens was similar. The U76-GJ75, that is, the reinforced UHPC column, had the largest confined strength, but underwent completely brittle failure. Compared with U0-GJ75, the obviously thicker UHPC cover led to a higher average confined stress. The enhancement mainly resulted from the larger UHPC area and stronger confinement for the inner NSC core. Moreover, the specimen with a thicker UHPC cover underwent a large sudden decrease. After the sudden decrease, the ring UHPC cover was split into multiple UHPC prisms connected through stirrups. Each UHPC prism could still carry the axial load. Thus, the specimen with a thicker UHPC cover had a larger residual confined stress after the sudden decrease. In the post-decrease stage, the confined stress of all the composite columns gradually decreased at a similar rate until failure.

Figure 7b shows the comparison of specimens with different volumetric stirrup ratios. For U25-GJ0, the complete removal of the stirrup accelerated the degradation of the confined stress in the post-decrease stage. The ultimate deformability of U25-GJ0 was obviously weaker, compared with that of other specimens. Additionally, U25-GJ75 delivered a visibly larger confined strength. Consistent with the classical confined concrete theory, a larger volumetric stirrup ratio resulted in higher confined strength. However, other specimens exhibited similar strain–stress relationships, including the confined strength and ductility. According to Zeng et al. (2020), the self-confinement from the UHPC may dominate the confining effect. Thus, a slight change in the stirrup did not have a large influence on the overall behavior. Moreover, the spacing between the stirrups may have been too large to provide an adequate confining effect. Considering the capacity of the loading setup, the specimens were scaled down, and required a higher volumetric stirrup ratio to maintain the confining effect compared with large-scale specimens. Thus, there was no obvious difference between U25-GJ100 to U25-GJ175.

The construction method effects are compared in Figure 7c. The specimens with a delay-casted inner NSC core had smaller confined strength. There may have been defects at the interface between the delay-casted inner NSC core and exterior UHPC cover. For the specimen with a delay-casted inner NSC core, the shrinkage of the exterior UHPC cover developed before pouring the inner NSC core. However, the shrinkage of the inner NSC core introduced defects at the UHPC-NSC interface. The confining effects from the UHPC cover and stirrup did not perfectly act on the inner NSC core. Thus, a slight reduction was obtained for U25-GJ75-L and U35-GJ75-L.

Table 4 summarizes the mechanical performance of all the specimens. The peak confined strength increased with the thickness of the exterior UHPC cover. Moreover, the linear correlation coefficient reached up to 0.956, which indicates a satisfactory linear relationship. Another interesting result is that all of the composite columns delivered similar compressive strain at the peak confined strength, which was approximately 3.0 × 10^−3^, and similar compressive strain at the peak strength of the plain UHPC. According to Zeng et al. (2020), the increased volumetric stirrup ratio rarely improved the deformability of the reinforced UHPC column under compression. Similarly, the ultimate deformability of the exterior UHPC cover in the composite column was not obviously extended by introducing the stirrup, regardless of the UHPC layer thickness. Thus, it can be concluded that the strain at the peak confined strength depended on the deformability of the plain UHPC.

The sudden decrease of the composite columns varied from 11.89 MPa to 49.57 MPa, which resulted in the rapid increase of the compressive strain varying from 0.02 × 10^−3^ to 0.45 × 10^−3^. The regularity of the sudden decrease was not very obvious, both in terms of confined stress and compressive strain. The descending modulus after the sudden decrease also exhibited negligible regularity with the thickness of the UHPC cover and stirrup ratio. First, the damage incurred by the exterior UHPC cover was considerably uncertain, with regard to the crack width and depth, development directions, and so on. Secondly, the crack of the UHPC cover may have disturbed the fully bonded interfacial behavior between the inner NSC core and exterior UHPC cover, which had very large dispersion and randomness. Thus, the confined stress and corresponding strain increment, and the subsequent descending modulus after the sudden decrease, had strong discreteness.

## 4. Modified Confined Model

**Figure 8:**
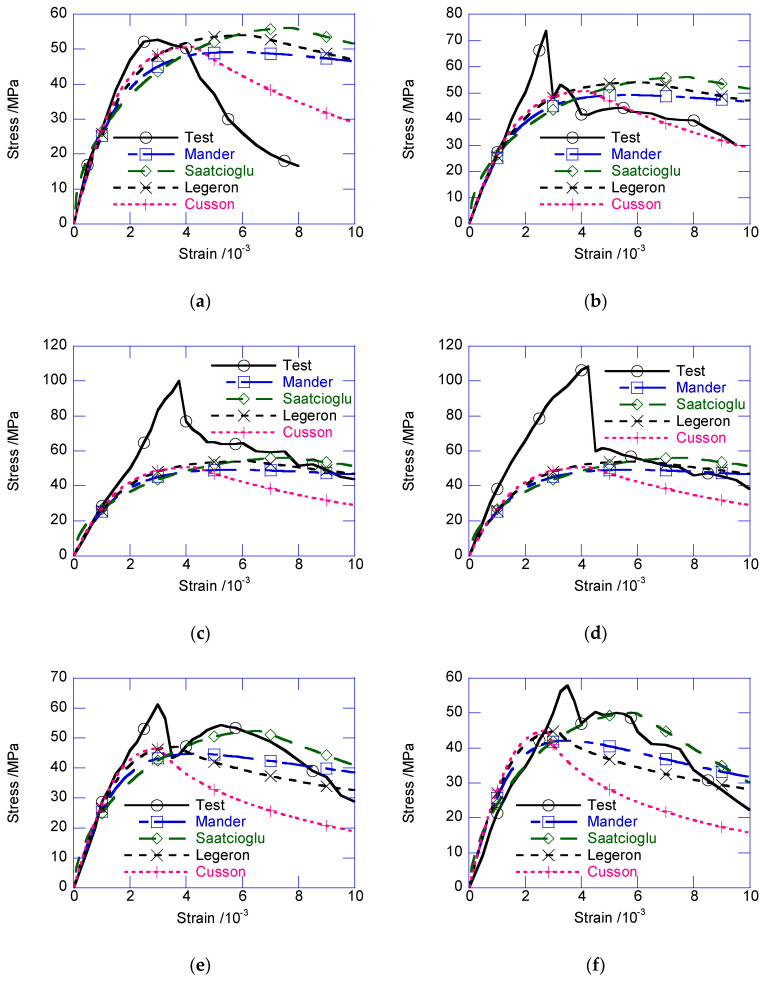

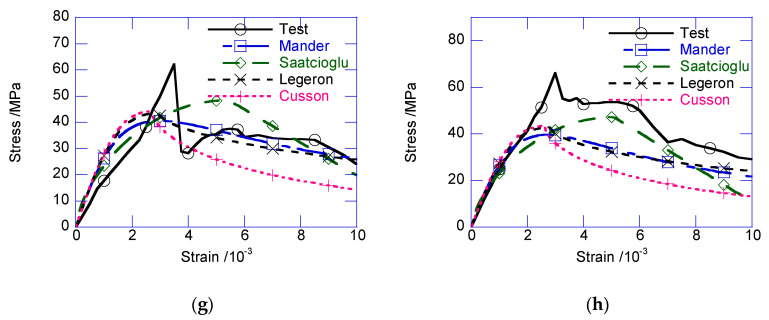
Comparison with classical reinforcement confined concrete model. (**a**) U0-GJ75; (**b**) U25-GJ75; (**c**) U30-GJ75; (**d**) U35-GJ75; (**e**) U25-GJ100; (**f**) U25-GJ125; (**g**) U25-GJ150; (**h**) U25-GJ175.

Figure 8 compares the strain–stress relationships with those of classical confined concrete models [14,15,16,17]. Note that, the confined stress of the composite column was the average stress, not specific for the NSC core or UHPC cover. According to the test results, before the peak confined stress, the test results delivered much higher confined stress than the prediction obtained using these models. The participation of the exterior UHPC cover in load-carrying significantly enhanced the peak confined strength. Moreover, the sudden decrease of the composite columns was reflected in the confined models.

**Figure 9:**
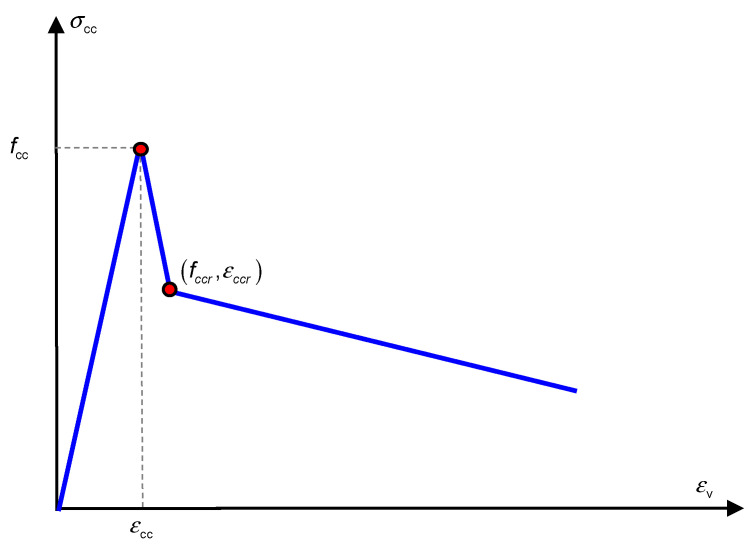
Modified confined model for reinforced UHPC-NSC composite column.

Based on these observations, a trilinear model was developed specifically for describing the confined stress of the reinforced UHPC-NSC composite column, as shown in Figure 9. The three parts were the linear ascending part, sudden decrease part, and ultimate deterioration part. The experimental strain–stress relationships ensured the rationality of the trilinear models. Two characteristic mechanical points should be addressed, namely, the peak point and the point after the sudden decrease.

First, the confined stress linearly developed to the confined strength *f*_cc_, which corresponds to strain $\varepsilon_{\mathrm{cc}}$; $\varepsilon_{\mathrm{cc}}$ can be approximately considered as the compressive strain of plain UHPC. Equation (4) was developed to estimate the peak confined strength *f*_cc_. Notably, Equation (4) contains an assumption whereby the confined strength of the exterior UHPC cover is rarely improved by the confining effect. According to Zeng et al. (2020), the confinement from the stirrup rarely contributes to the strength of the UHPC material. Thus, the stress of the exterior UHPC cover can be considered the same as that of the material compressive test.

(4) $$f_{\mathrm{cc}}=\mu_{U}f_{\mathrm{co}\_U}+\mu_{N}(f_{\mathrm{co}\_N}+\lambda f_{l})$$

where *f*_co_U_ and *f*_co_N_ are the compressive strengths of the plain UHPC and NSC, respectively, as presented in Table 3; $\mu_{U}$ and $\mu_{N}$ are the area ratio of the UHPC and NSC to the total confined area, respectively, as expressed in Equation (5); *f*_l_ is the confining stress and λ is the confining coefficient, which must be different to the confined NSC column when using UHPC-NSC composite columns.

(5) $$\mu_{U}=1-\mu_{N},\mu_{N}=\frac{d-2t_{u}}{d}$$

**Figure 10:**
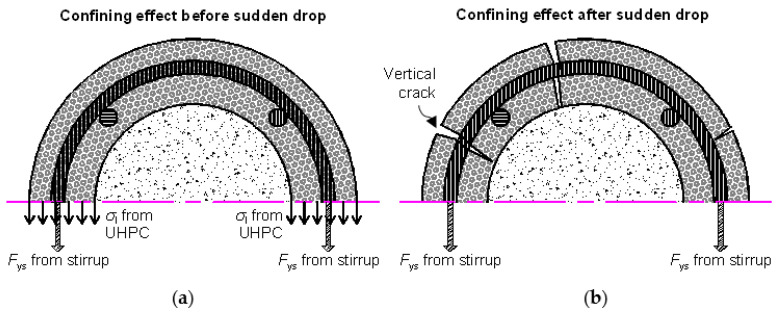
Confinement from exterior UHPC cover. (**a**) Confining effect before sudden drop; (**b**) confining effect after sudden drop.

The confining stress *f*_l_ is described on the left side of Figure 10. At the peak confined strength *f*_cc_, the stirrup and UHPC cover provided the confining stress at the same time. At the peak confined strength, the stirrup strain was considered to have been subjected to the ultimate tensile strain of the UHPC, the thickness of the cover layer was ignored, and all UHPC materials achieved the ultimate tensile strength *f*_tu_ [5]. Thus, *f*_l_ can be calculated as follows:

(6) $$f_{l}=\frac{\pi d_{s}^{2}E_{s}\varepsilon_{\mathrm{tu}}}{2sc}+\frac{2f_{\mathrm{tu}}t_{u}}{c}$$

**Figure 11:**
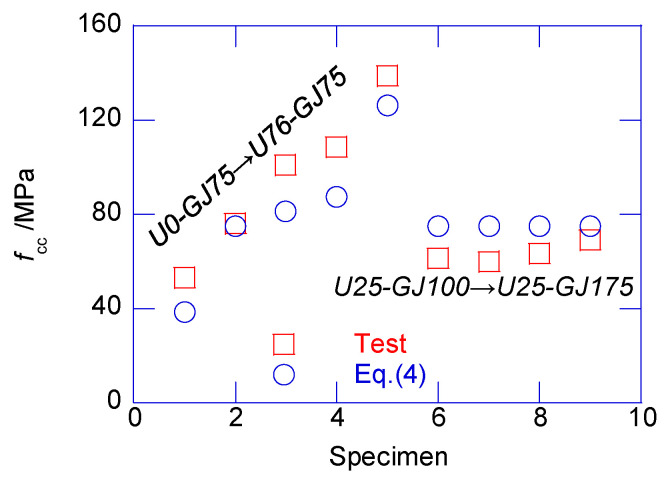
Estimation of peak confined strength *f*_cc_ of composite columns.

The confining coefficient λ was derived by regression analysis as λ=3.128. Figure 11 shows the comparison between the test results and the prediction made using Equation (4). The average prediction error was only −0.2%, demonstrating the acceptable accuracy.

**Figure 12:**
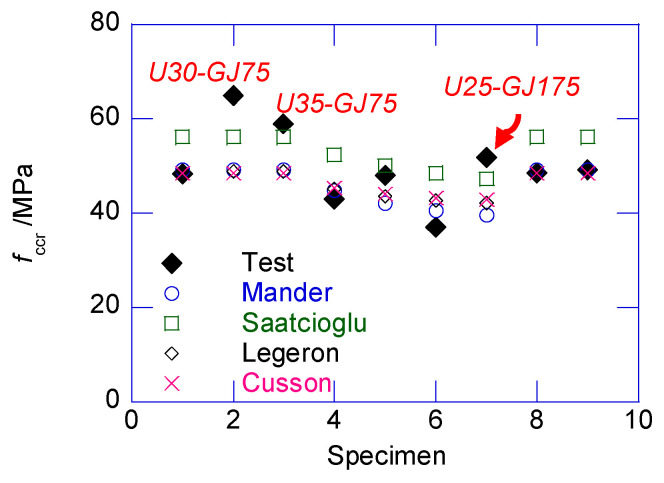
Comparison of confined stress after sudden decrease *f*_ccr_ and prediction.

In the post-peak stage, the strain–stress relationship returned to the covered range of the classic models. The confined stress after the sudden decrease only approximately agreed with the peak confined strength predicted by several classical models. After the sudden decrease, the circumferential tensile stress in the UHPC cover disappeared. The cracked UHPC cover, which existed as multiple independent UHPC prisms, bonded with the inner NSC core, which transferred the confining stress from the stirrup. At this stage, the UHPC prisms can be considered the NSC material, owing to the negligible circumferential tensile strength. Thus, the confined strength after the sudden decrease can be considered as the peak confined strength of the NSC column. By ignoring the tensile strength of UHPC cover, and substituting the design parameters in the classic confined NSC models, such as the Mander, Saatcioglu, Legeron, Cusson models, the post-drop strength of the composite column can be derived, as presented in Figure 12. The average prediction errors of the Mander, Saatcioglu, Legeron, Cusson models are −4.98%, 8.10%, −17.26%, −4.86% respectively. The Mander model delivered the best prediction. Except for U30-GJ75, U35-GJ75, and U25-GJ175, after the sudden decrease, the confined stress was located in the cover of several classical models. After the sudden decrease, the compressive strain $\varepsilon_{\mathrm{ccr}}$ was approximated using Equation (7), where 0.23 × 10^−3^ is the average value of $\varepsilon_{\mathrm{ccr}}-\varepsilon_{\mathrm{cc}}$ obtained in this test.

(7) $$\varepsilon_{\mathrm{ccr}}=\varepsilon_{\mathrm{cc}}+0.23\times 10^{-3}$$

With regard to the ultimate deteriorated part, it can be accepted that the average descending modulus can be considered as the slope of the third straight part, which was equal to −3.43 GPa in this study. Considering the significant discreteness and randomness of the UHPC compressive damage, and the impossibility of the post-damage condition in the structural service, the approximations for *f*_ccr_, $\varepsilon_{\mathrm{ccr}}$, and *E*_sd_ are acceptable. Further sophisticated tests must be conducted to investigate the strong nonlinear behavior of this composite column.

## 5. Conclusions

This study focused on the axial behavior of a UHPC-NSC composite column under compression. Twelve specimens were tested to investigate the effect of different design parameters, namely, the thickness of the UHPC cover, volumetric stirrup ratio, and construction method. The failure modes and strain–stress relationships are discussed in this paper. Unlike the reinforced NSC column and UHPC column, the composite column underwent a distinctive sudden decrease immediately after the peak confined strength. A trilinear model is proposed to describe the strain–stress relationship of the reinforced UHPC-NSC composite column, and it was found to be in good agreement with the test results. The major conclusions drawn from this study and future research directions are as follows:

1. The peak confined strength of the reinforced UHPC-NSC composite column occurred at the compressive strain of the UHPC material. The confinement from the stirrup rarely enhanced the deformation of the exterior UHPC cover.
2. The distinctive sudden decrease was caused by the loss of confining stress from the exterior UHPC cover. After the sudden decrease, the mechanical status of the composite column degenerated into the reinforced NSC column, wherein the confining stress was mainly provided by the stirrup.
3. The confinement from the stirrup did not contribute to the compressive strength of the exterior UHPC cover, and the proposed trilinear model achieved acceptable accuracy, compared with the test results.
4. Due to the excessively large spacing of stirrups in the small scaled specimens, the confinement from stirrup was inadequate. Further sophisticated tests for the reinforced UHPC-NSC composite column with the consideration of size effect must be conducted to independently investigate the load sharing and deformation status.

## Figures and Tables

**Table 1:** Parameters of specimens.

Specimen	Type	*t*_u_/mm	*s*/mm	*ρ*_v_	Remark
U0-GJ75	NSC	0	75	1.26%	Benchmark
U25-GJ75	Composite	25	75	1.26%	Thickness of UHPC cover
U30-GJ75		30	75	1.26%	
U35-GJ75		35	75	1.26%	
U76-GJ75	UHPC	-	75	1.26%	
U25-GJ100	Composite	25	100	0.94%	Volumetric stirrup ratio
U25-GJ125		25	125	0.76%	
U25-GJ150		25	150	0.63%	
U25-GJ175		25	175	0.54%	
U25-GJ0		25	-	0.0%	
U25-GJ75-L		25	75	1.26%	Construction method
U35-GJ75-L		35	75	1.26%	

Remark: *t*_u_ is the thickness of exterior UHPC cover; *s* is the spacing between adjacent stirrups in middle area; *ρ*_v_ is the representative volumetric stirrup ratio.

**Table 2:** UHPC Ingredients.

Matrix (kg/m^3^)	Performance Indicators of Steel Fibers				
Cement: 680 Steel fiber: 100 Water: 160 Concrete Admixtures: 18	Volume ratio	Length (mm)	Density (kg/m^3^)	Diameter (μm)	Tensile modulus of elasticity (Gpa)
	2%	12	0.78	20	200

**Table 3:** Mechanical properties of UHPC and NSC.

Material	*E*/GP*a*	*f*_ti_/MPa	*f*_tu_/MPa	$\varepsilon_{\mathrm{tu}}$/10^−3^	*f*_co_/MPa	$\varepsilon_{\mathrm{co}}$/10^−3^
UHPC	43.38	7.8	9.29	1.08	126.45	3.08
NSC	29.51	-	-	-	38.63	2.01

Remark: *E* is the elastic modulus; *f*_ti_ is the initial cracking strength; *f*_tu_ is the peak tensile strength; $\varepsilon_{\mathrm{tu}}$ is the tensile strain at peak tensile strength; *f*_co_ is the peak compressive strength; $\varepsilon_{\mathrm{co}}$ is the compressive strain at peak compressive strength.

**Table 4:** Mechanical performance of specimens.

Specimen	*f*_cc_/MPa	$\varepsilon_{\mathrm{cc}}/10^{-3}$	*f*_ccr_/MPa	$\varepsilon_{\mathrm{ccr}}/10^{-3}$	(*f*_cc_−*f*_ccr_)/MPa	$(\varepsilon_{\mathrm{ccr}}-\varepsilon_{\mathrm{cc}})/10^{-3}$	*E*_sd_/GPa
U0-GJ75	53.20	2.76	-	-	-	-	-
U25-GJ75	76.16	2.88	48.27	2.90	27.89	0.02	−2.44
U30-GJ75	101.04	3.82	65.08	4.07	35.96	0.25	−4.71
U35-GJ75	108.80	4.20	59.23	4.47	49.57	0.27	−3.91
U76-GJ75	138.63	3.26	-	-	-	-	-
U25-GJ100	61.34	2.98	43.08	3.43	18.26	0.45	−2.73
U25-GJ125	59.90	3.56	48.01	3.81	11.89	0.25	−4.85
U25-GJ150	63.43	3.47	37.08	3.57	26.35	0.10	−0.71
U25-GJ175	69.06	3.15	51.89	3.29	17.17	0.14	−4.50
U25-GJ0	67.77	2.66	43.04	2.67	24.73	0.01	−4.52
U25-GJ75-L	72.99	3.67	48.46	3.71	24.53	0.04	−2.75
U35-GJ75-L	97.43	3.27	49.13	4.13	48.30	0.86	−3.16

Remark: *f*_cc_ is the peak confined strength; $\varepsilon_{\mathrm{cc}}$ is the compressive strain at peak confined strength *f*_cc_; *f*_ccr_ is the confined strength after the sudden drop; $\varepsilon_{\mathrm{ccr}}$ is the compressive strain at confined strength *f*_ccr_; *E*_sd_ is the regressed descending modulus from the sudden drop to the failure of specimen.
